# NADPH Oxidase 4-Derived H_**2**_O_**2**_ Promotes Aberrant Retinal Neovascularization via Activation of VEGF Receptor 2 Pathway in Oxygen-Induced Retinopathy

**DOI:** 10.1155/2015/963289

**Published:** 2015-03-18

**Authors:** Jingming Li, Joshua J. Wang, Sarah X. Zhang

**Affiliations:** ^1^The First Affiliated Hospital of Xi'an Jiaotong University College of Medicine, 277 West Yanta Road, Xi'an, Shaanxi 710061, China; ^2^Department of Medicine, Endocrinology and Diabetes, Harold Hamm Oklahoma Diabetes Center, University of Oklahoma Health Sciences Center, 941 Stanton L. Young Boulevard, Oklahoma City, OK 73104, USA; ^3^Department of Ophthalmology & Ira G. Ross Eye Institute, School of Medicine and Biomedical Sciences, University at Buffalo, The State University of New York, Buffalo, NY 14214, USA; ^4^SUNY Eye Institute, Buffalo, NY 14214, USA

## Abstract

NADPH oxidase 4 (Nox4) is a major isoform of NADPH oxidase in retinal endothelial cells. Our previous study suggests that upregulation of Nox4 in retinal endothelial cells contributes to retinal vascular leakage in diabetes. In the current study, we investigated the role and mechanism of Nox4 in regulation of retinal neovascularization (NV), a hallmark of proliferative diabetic retinopathy (PDR), using a mouse model of oxygen-induced retinopathy (OIR). Our results confirmed that Nox4 was expressed predominantly in retinal vasculature of mouse retina. Retinal expression of Nox4 was markedly increased in OIR, in parallel with enhanced phosphorylation of ERK. In human retinal microvascular endothelial cells (HRECs), overexpression of Nox4 by adenovirus significantly increased extracellular H_2_O_2_ generation, resulting in intensified VEGFR2 activation and exacerbated angiogenesis upon VEGF stimulation. In contrast, silencing Nox4 expression or scavenging H_2_O_2_ by polyethylene glycol- (PEG-) conjugated catalase inhibited endothelial migration, tube formation, and VEGF-induced activation of VEGFR2 signaling. Importantly, knockdown of retinal Nox4 by adenovirus-delivered siRNA significantly reduced ERK activation and attenuated retinal NV formation in OIR. Taken together, our data indicate that Nox4 promotes retinal NV formation through H_2_O_2_/VEGFR2/ERK signaling pathway. Reducing retinal Nox4 expression may represent a promising therapeutic approach for neovascular retinal diseases such as PDR.

## 1. Introduction

Aberrant retinal neovascularization (NV) is a leading cause of visual impairment and blindness in neovascular eye diseases such as retinopathy of prematurity (ROP), diabetic retinopathy (DR), and age-related macular degeneration (AMD). Newly developed anti-VEGF therapies have shown promise in short-term treatment; however, their long-term efficacy in retinal NV and retinal function remains uncertain. A better understanding of the mechanisms underlying retinal NV and identifying novel therapeutic targets are desperately needed for the development of new strategies to prevent and treat neovascular retinal diseases.

Vascular endothelial growth factor (VEGF) is generally accepted as the most potent inducer of endothelial activation and angiogenesis, a process where new vessels develop from preexisting vasculature. In normal retina, VEGF was found mainly to be expressed in retinal neurons and glial cells and to exist scarcely in blood vessels [1, 2]. Under ischemic condition, as seen in many neovascular diseases, retinal expression and production of VEGF is drastically increased [3]. Through a paracrine mechanism, VEGF binds to its cell-surface receptors, including VEGFR1/Flt-1, VEGFR2/Flk-1/KDR, and VEGFR3, promoting endothelial cell survival, proliferation, migration, and tubular structure formation [2]. Among these receptors, VEGFR1 and VEGFR2 are predominantly expressed by vascular endothelial cells and VEGFR3 is mainly found in lymphatic endothelial cells. Activation of VEGFR1 plays a dual role in either stimulating or inhibiting angiogenesis, while activation of VEGFR2 is believed to promote angiogenesis [4, 5]. Furthermore, VEGF-induced endothelial mitogenesis and permeability are mainly mediated by VEGFR2 [6]. Upon binding to VEGF, VEGFR2 undergoes dimerization and autophosphorylation, resulting in activation of its downstream kinases including mitogen-activated protein kinase (MAPK) (ERK1/2, p38, JNK), PI3K/Akt, and endothelial nitric oxide synthase (eNOS), which may further lead to alterations in endothelial cell survival, proliferation, and migration.

Reactive oxygen species (ROS) have been implicated in the development of neovascular retinal diseases [7, 8]. Low or moderate levels of ROS act as a signal transducer that stimulates angiogenesis [9], while excessive ROS can cause endothelial dysfunction and apoptosis resulting in loss of vascular cells and ischemia, which in turn triggers blood vessel growth [10]. Thus ROS generation is closely associated, directly or indirectly, with pathological NV formation in neovascular retinal diseases such as ROP [11]. One major source of ROS in endothelial cells is NADPH oxidase. The family of NADPH oxidase consists of 7 isoforms: Nox1–5, Duox1, and Duox2. Among these isoforms, Nox4 is unique in that it is constitutively active and primarily generates H_2_O_2_ instead of superoxide [12]. Previous studies showed that Nox4 was prominently expressed in new capillaries in ischemic brain tissue [13]. Enhancing endothelial Nox4 expression accelerates recovery from hindlimb ischemia [14] and deficiency of Nox4 attenuates angiogenesis after femoral artery ligation [15]. These results suggest that Nox4 contributes to tissue ischemia-induced angiogenic response. We previously reported that Nox4 is a major isoform of NADPH oxidase in retinal endothelial cells and its expression is upregulated by diabetes. Inhibition of Nox4 ameliorates blood-retinal barrier (BRB) breakdown and retinal vascular leakage in diabetic animals through a VEGF-dependent mechanism [16]. However, the role of Nox4 in the pathogenesis of retinal NV, another hallmark of DR, remains largely unknown.

In the present study, we investigated the role of Nox4 in retinal angiogenesis and its contribution to retinal NV formation in a mouse model of OIR. Our results suggest that Nox4 is potentially implicated in retinal vasculature development and contributes to aberrant blood vessel growth in neovascular retinal diseases through regulation of the VEGF/VEGFR2 pathway.

## 2. Materials and Methods

### 2.1. Experimental Animals

The mouse model of OIR was set up as described [17, 18]. All animal studies were carried out in accordance with Association for Research in Vision and Ophthalmology (ARVO) Statement for Use of Animals in Ophthalmic and Vision Research and University of Oklahoma Health Sciences Center (OUHSC) Guideline for Animal in Research.

### 2.2. Adenoviral Amplification, Purification, and Titration

Ad-Nox4i and Ad-Ctrli were kindly provided by Dr. Kai Chen [19] and Ad-Nox4 and Ad-LacZ were gifts from Dr. Mahadev et al. [20]. Adenoviral vectors were amplified, purified, and titrated as described previously [16].

### 2.3. Periocular Injection

Periocular injection of adenoviral vector was performed at P12 mouse pups. One eye received periocular injection of purified adenoviral vector of Ad-Nox4i at dose of 10^10^ viral particles and contralateral eye was injected with the same amount of Ad-Ctrli as control.

### 2.4. Quantification of Retinal Neovascularization

Retinal neovascularization was quantified by counting vascular cell nuclei on the vitreal side of internal limiting membrane as described before [18]. For retinal angiography, PBS containing high molecular weight FITC-dextran (Sigma-Aldrich, St. Louis, MO) was injected into the left ventricle of anesthetized mouse pups. Eyes were enucleated and retinas were flat-mounted. Retinal NV was measured by Adobe Photoshop software as reported [17].

### 2.5. Immunostaining

Eyeballs were embedded in OCT and sectioned by Leica cryostat. After blocking, sections were incubated with anti-Nox4 primary antibody (Santa-Cruz Biotechnology Inc., Santa Cruz, CA), anti-CD31 (BD Pharmingen, San Diego, CA), or anti-p-ERK (Cell Signaling Technology, Boston, MA) overnight. Signals were detected with Alexa Fluor 488 or Alexa Fluor 594 conjugated secondary antibodies (Invitrogen, Carlsbad, CA). Slides were mounted with VECTASHIELD mounting medium with DAPI (Vector Laboratories, Burlingame, CA) and visualized under fluorescence microscope (Olympus). Sections with omission of primary antibodies were used as negative controls. For retinal flat-mount staining, retinas were dissected out and permeabilized followed by incubation with anti-Nox4 antibody (Santa-Cruz) and anti-CD31 (BD Pharmingen). After intensive rinse, retinas were stained with Alexa Fluor 488 and Alexa Fluor 594 conjugated secondary antibody (Invitrogen). The retinas were flat-mounted and observed under fluorescence microscope (Olympus).

### 2.6. Cell Culture

Primary human retinal microvascular endothelial cells (HRECs) were purchased from Cell Systems Inc. (Kirkland, WA) and cultured as described previously [16]. Subconfluent HRECs were transduced by adenovirus at a multiplicity of infection (MOI) 20 for 48 h. After being quiescent in EBM with or without 1000 U/mL polyethylene glycol/catalase (PEG-Catalase, Sigma-Aldrich) for 24 h, cells were treated with recombinant human VEGF_165_ (PeproTech, Inc., Rocky Hill, NJ) for indicated time and subjected to biochemical assays.

### 2.7. Real-Time RT-PCR

After RNA was extracted and first-stand cDNA was synthesized, real-time RT-RCR was performed as described [16]. Primer sequences for real-time RT-PCR were Nox4, forward: 5′-ACT TTT CAT TGG GCG TCC TC-3′ and reverse: 5′-AGA ACT GGG TCC ACA GCA GA-3′; CD31, forward: 5′-AGG CTT GCA TAG AGC TCC AG-3′ and reverse: 5′-TTC TTG GTT TCC AGC TAT GC-3′. Relative mRNA level was calculated by the ΔΔCt method using 18 s as control.

### 2.8. Western Blot Analysis

Retinal samples were prepared as described previously [16]. Twenty-five-microgram protein was subjected to SDS-PAGE and blotted to nitrocellulose membranes. Membranes were with anti-Nox4 antibody (Santa-Cruz), anti-p-VEGFR2, anti-p-ERK1/2 antibody, anti-ERK1/2 antibody, anti-p-P38 antibody, and anti-P38 MAPK antibody (Cell Signaling), followed by incubation with HRP-conjugated secondary antibodies (Vector Laboratories). The same membrane was reblotted with the anti-*β*-actin antibody (Sigma-Aldrich) as loading control.

### 2.9. Tube Formation Assay

35 mm culture dishes (BD Biosciences) were coated with 500 *μ*L Matrigel Basement Membrane Matrix (BD Biosciences) for 30 min at 37°C. Adenoviral transduced HRECs were dissociated by Cellstripper (Mediatech, Inc., Manassas, VA) and resuspended in EBM. Then HRECs (10^5^) were seeded on Matrigel Basement Membrane Matrix. After 6 h or 16 h of incubation, branching numbers of tubes were quantified in 3 random visual fields under light microscope (Olympus).

### 2.10. Transwell Migration Assay and Wound Healing Assay

HRECs transwell migration assay was performed on semipermeable membranes (Costar Transwell, Corning, NY) as described [21]. For wound healing assay, confluent HRECs were wounded with 1 mL pipette tips and allowed to migrate in growth medium. After 16 h, migration was quantified by Image J analysis software (NIH, Bethesda, MD).

### 2.11. Detection of Extracellular Hydrogen Peroxide Generation

Extracellular hydrogen peroxide was determined by Amplex red (Invitrogen) as described [16].

### 2.12. Statistical Analysis

Data were presented as mean ± SD. Statistical comparisons were performed by using ANOVA with Bonferroni's post hoc test. *P* value less than 0.05 was considered as statistically significant.

## 3. Results

### 3.1. Localization of Nox4 to Retinal Blood Vessels and Expression of Nox4 in OIR

**Figure 1:**
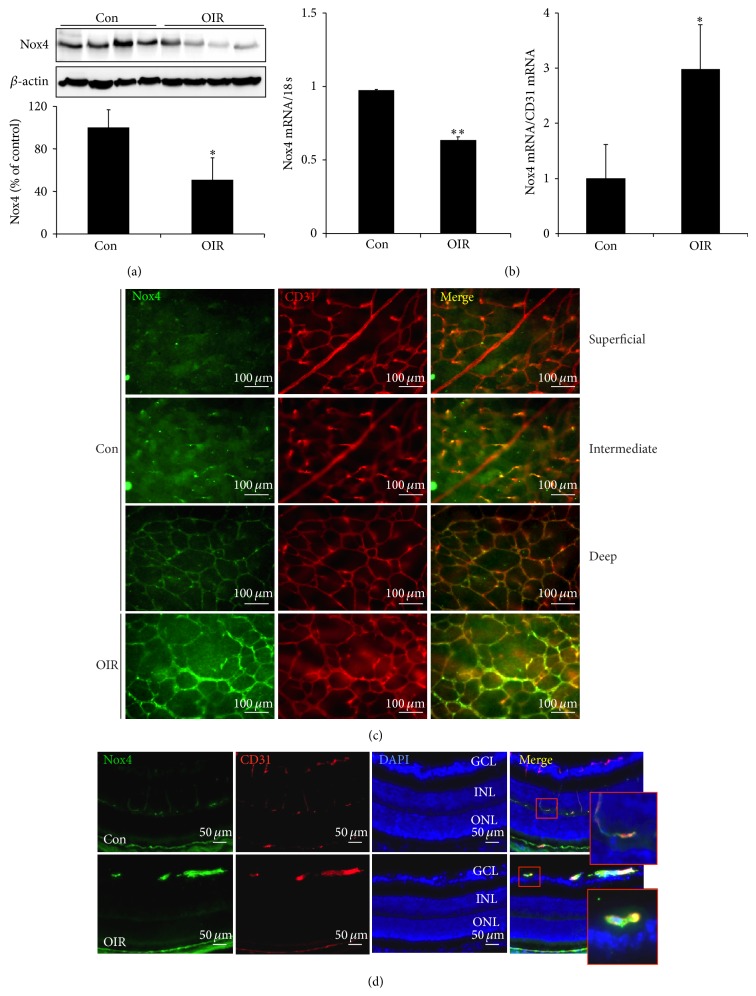
Nox4 expression in retinas from OIR and control mice at P12. (a) Expression of Nox4 protein in the retina in P12 OIR and control mice was determined by Western blot analysis and semiquantified by densitometry. *n* = 4 for each group. (b) Expression of Nox4 mRNA in the retina in P12 OIR and control mice was determined by real-time RT-PCR and normalized to 18 s (left panel) or to CD31 (right panel). *n* = 4 for control group and *n* = 6 for OIR group. ^*^ *P* < 0.05 or ^**^ *P* < 0.01 versus Con. (c) Representative images of retinal whole mounts from P12 control mice stained with anti-Nox4 antibody (green) and anti-CD31 antibody (red). (d) Cryosections of eyeballs from P12 OIR and control mice were stained with anti-Nox4 antibody (green) and anti-CD31 antibody (red).

To induce OIR, newborn pups of postnatal day 7 (P7) with their feeding mother were exposed to 75 ± 1% oxygen for 5 days, which results in significant loss of blood vessels in central area of the retina, or vasoobliteration. On P12, pups were returned to room air (RA). The alteration in oxygen concentration produces a relative hypoxia in the retina, which triggers new blood vessel growth (NV) that peaks at P17 [17, 18]. To determine the effect of hyperoxia on retinal Nox4 expression, we measured the protein and mRNA levels of Nox4 in the retina of P12 OIR mice. Retinas were dissected from mice immediately after oxygen treatment and subjected to Western blot and real-time PCR analyses. As shown in Figures 1(a) and 1(b) (left panel), both protein and mRNA levels of Nox4 were significantly lower in OIR when compared to age-matched controls. Interestingly, mRNA level of Nox4, if normalized by CD31, a specific marker of endothelial cells, shows a significant increase in OIR (Figure 1(b), right panel). Since loss of blood vessels is a hallmark of retinal changes in P12 OIR mice, we suspected that the reduction in Nox4 expression may be associated with the loss of blood vessel content in OIR retinas. Using immunofluorescence approach, we confirmed Nox4 expression in retinal blood vessels. Immunostaining of P12 retinal whole mounts shows that Nox4 is expressed mainly in the deep layer of retinal capillaries colocalizing with endothelial cell marker CD31; only weak signals of Nox4 were detected in superficial and intermediate layers of retinal blood vessels (Figure 1(c)). In P12 OIR retina, the overall staining of Nox4 was reduced in parallel with vasoobliteration; however, much higher level of Nox4 immunoreactivity was observed in remnant retinal blood vessels (Figure 1(d)), which is consistent with the results in Figure 1(b).

**Figure 2:**
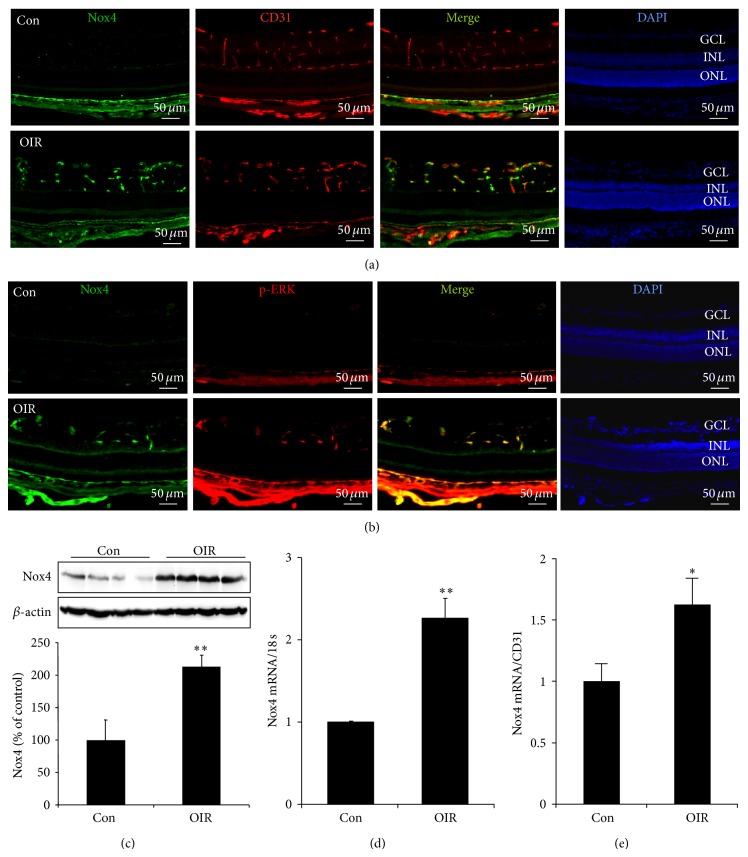
Nox4 expression in retinas of OIR mice at retinal NV phase. (a) Immunostaining of Nox4 (green) in retinal cryosections from P17 OIR and control mice. CD31 (red) was used to label retinal vessels. (b) Expression of phosphorylated ERK (red) and its colocalization with Nox4 (green) in the retina in P17 OIR and control mice were determined by immunostaining. (c) Expression of Nox4 protein in the retina in P15 OIR and control mice was determined by Western blot analysis and semiquantified by densitometry. *n* = 4 for each group. ^**^ *P* < 0.01 versus Con. (d-e) mRNA of Nox4 in the retina in P15 OIR and control mice was measured by real-time RT-PCR and normalized to 18 s (d) or to CD31 (e). *n* = 4 for control group and *n* = 6 for OIR group. ^**^ *P* < 0.01 versus Con.

In P15 and P17 OIR retina, intensive staining of Nox4 was observed in both intrinsic retinal vasculature and preretinal new blood vessels (Figure 2(a)). Moreover, signals of Nox4 colocalized with enhanced phosphorylation of ERK (Figure 2(b)). Increased Nox4 protein and mRNA levels were further confirmed by Western blot analysis (Figure 2(c)) and real-time PCR (Figures 2(d) and 2(e)), respectively. Upregulation of Nox4 in OIR suggests a potential role of Nox4 in retinal NV formation.

### 3.2. Overexpression of Nox4 Promotes Retinal Endothelial Tube Formation and Potentiates VEGF-Dependent VEGFR2 Signaling

**Figure 3:**
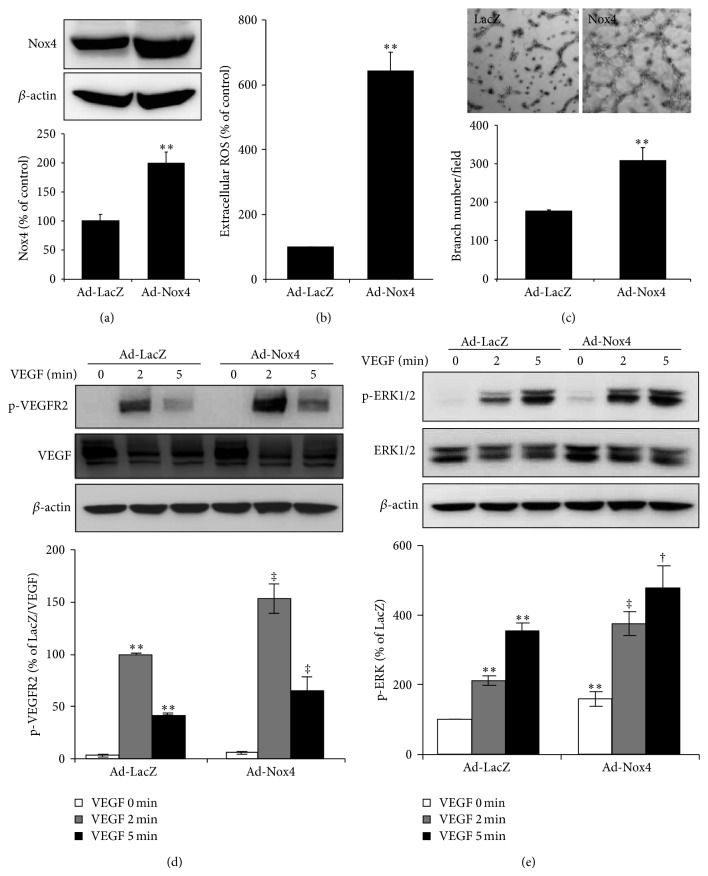
Overexpression of Nox4 promotes endothelial tube formation and augments VEGFR2 activation in HRECs. HRECs were transduced with Ad-LacZ and Ad-Nox4 for 48 h and subjected to biochemical and functions analyses. (a) Expression of Nox4 was determined by Western blot analysis and semiquantified by densitometry. *n* = 3; ^**^ *P* < 0.01 versusAd-LacZ. (b) Generation of extracellular ROS was measured by Amplex red assay. *n* = 3; ^**^ *P* < 0.01 versus Ad-LacZ. (c) Tube formation of HRECs capacity was evaluated by Matrigel assay. Adenoviral-transduced HRECs were seeded on Matrigel for 6 h and tube numbers were counted from 3 random visual fields. ^**^ *P* < 0.01 versus Ad-LacZ. Results represent 3 independent experiments. (d-e) Phosphorylation of VEGFR2 (d) and ERK (e) induced by VEGF (50 ng/mL) was determined by Western blot analysis and semiquantified by densitometry. *n* = 4; ^**^ *P* < 0.01 versus Ad-LacZ and ^†^ *P* < 0.05 or ^‡^ *P* < 0.01 versus Ad-LacZ with VEGF.

Previously we demonstrated that hypoxia, a potent inducer of endothelial activation and angiogenesis, upregulates Nox4 mRNA and protein expression in human retinal endothelial cells (HRECs) [16]. To investigate if Nox4 regulates the angiogenic activity of retinal endothelial cells, we overexpressed Nox4 in HRECs and examined its effect on endothelial tube formation. Expression levels of Nox4 in adenoviral transduced HRECs were confirmed by Western blot analysis (Figure 3(a)). Overexpression of Nox4 increased extracellular ROS generation in HRECs by more than 6-fold (Figure 3(b)) and significantly promoted endothelial tube formation (Figure 3(c)). Because VEGFR2 activation is considered to be a critical step in VEGF-induced endothelial angiogenic response, we investigated the role of Nox4 in VEGFR2 signaling in HRECs. As shown in Figure 3(d), VEGF induced a robust phosphorylation of VEGFR2 at Tyr 1175, which was further enhanced by Nox4. Additionally, overexpression of Nox4 augmented VEGF-induced activation of ERK1/2-MAPK pathway (Figure 3(e)), a downstream effector of VEGFR2 signaling. These results indicate that upregulation of Nox4 may promote angiogenic response in retinal endothelial cells through regulation of the VEGF-R2/ERK-MAPK pathways.

### 3.3. Knockdown of Nox4 Attenuates VEGF-Induced VEGFR2 Activation, Endothelial Cell Migration, and Tube Formation

**Figure 4:**
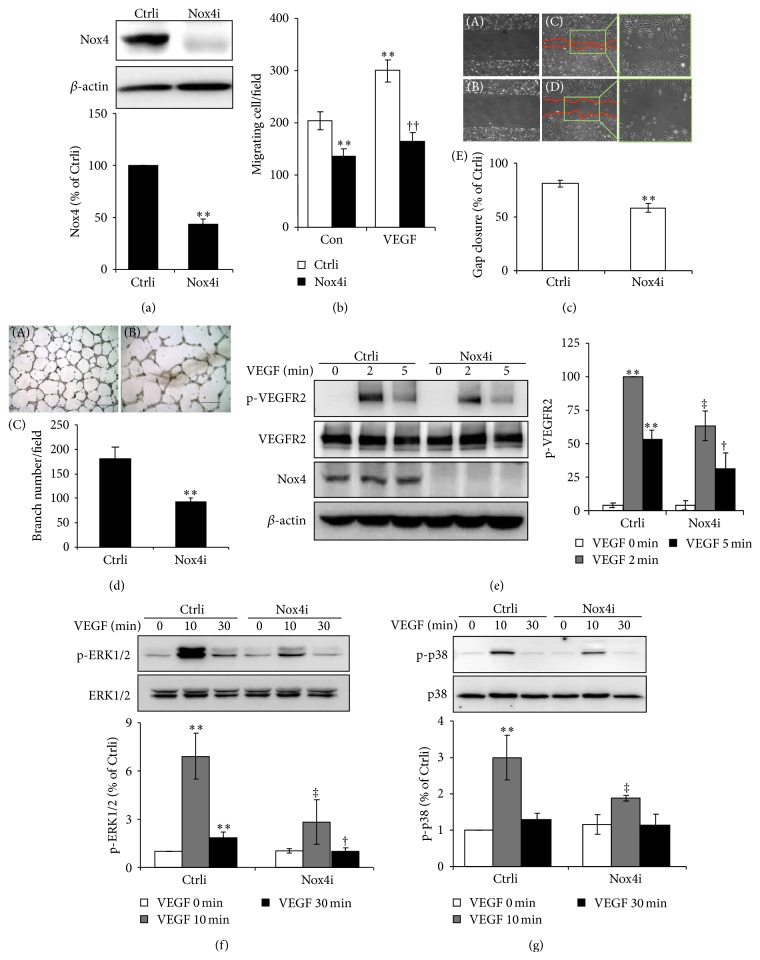
Knockdown of Nox4 in HRECs suppresses endothelial angiogenic response and inhibits VEGFR2 signaling pathway. HRECs were transduced with Ad-Ctrli and Ad-Nox4i for 48 h. (a) Nox4 expression in HRECs was determined by Western blot analysis and semiquantified by densitometry. *n* = 3; ^**^ *P* < 0.01 versus Ctrli. (b-c) Migration of HRECs was measured by transwell migration assay (b) and wound healing assay (c). Migrated cell number or gap closure was calculated from 3 different wells. ^**^ *P* < 0.01 versus Ctrli and ^‡^ *P* < 0.01 versus Ctrliwith VEGF. Results represent 3 independent experiments. (d) Endothelial tube formation capacity was evaluated by Matrigel assay. HRECs were seeded on Matrigel for 16 h and tube numbers were counted from 3 random visual fields. (d-A) HRECs with Ad-Ctrli, (d-B) HRECs with Ad-Nox4i, and (d-C) quantification results. ^**^ *P* < 0.01 versus Ctrli. Results represent 3 independent experiments. (e–g) Phosphorylation of VEGFR2 (e), ERK (f), and P38 (g) induced by VEGF (50 ng/mL) was determined by Western blot analysis and semiquantified by densitometry. *n* = 4; ^**^ *P* < 0.01 versus Ctrli and ^†^ *P* < 0.05 or ^‡^ *P* < 0.01 versus Ctrli with VEGF.

Endothelial migration is an initial step in angiogenesis. To determine whether VEGF-induced endothelial migration requires Nox4, we knocked down Nox4 by transducing HRECs with adenovirus mediated Nox4 RNAi (Ad-Nox4i) or control RNAi (Ad-Ctrli) and then evaluated endothelial migration in the presence of VEGF. As shown in Figure 4(a), expression of Nox4 was significantly reduced in HRECs transduced with Ad-Nox4i. These cells demonstrated impaired capacity in both basal and VEGF-stimulated migration as analyzed by transwell assay (Figure 4(b)) and “wound healing” assay (Figure 4(c)). Furthermore, knockdown of Nox4 significantly blocked retinal endothelial tube formation in response to VEGF stimulation (Figure 4(d)).

To determine whether Nox4 downregulation suppresses tube formation through suppressing VEGFR2 signaling, we examined VEGFR2 activation in HRECs induced by VEGF and its downstream signaling pathways such as ERK1/2-MAPK and p38-MAPK. As shown in Figures 4(e) and 4(f), knockdown of Nox4 in HRECs significantly attenuated VEGF-stimulated VEGFR2 phosphorylation and, likewise, reduced the phosphorylation of ERK1/2 and p38 MAPKs. These results have implied a role of Nox4 in VEGFR2-dependent angiogenic signaling in retinal endothelial cells.

### 3.4. Scavenging Nox4-Derived H_*2*_O_*2*_ Suppresses VEGF Signaling Pathway and Retinal Endothelial Tube Formation

**Figure 5:**
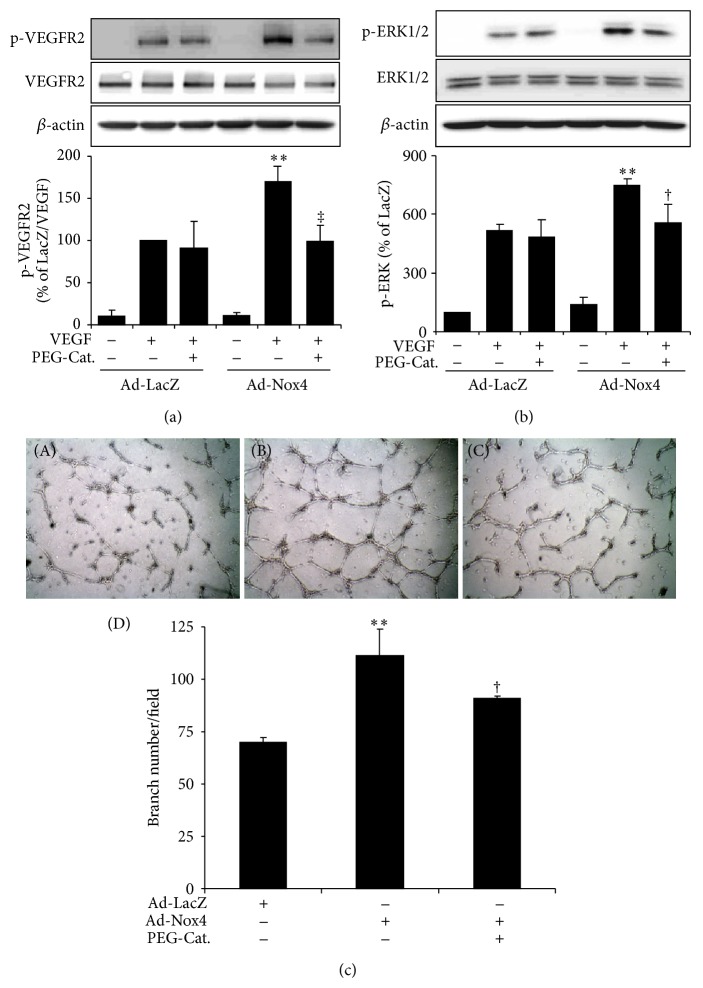
Scavenging of Nox4-derived H_2_O_2_ attenuated VEGFR2 activation and reduced endothelial tube formation. HRECs were transduced with Ad-LacZ and Ad-Nox4 for 48 h and then incubated with PEG-Catalase at dose of 1000 U/mL for 16 h. (a-b) Phosphorylation of VEGFR2 (a) and ERK (b) induced by VEGF (50 ng/mL) was determined by Western blot analysis and semiquantified by densitometry. *n* = 4; ^**^ *P* < 0.01 versus Ad-LacZ with VEGF and ^†^ *P* < 0.05 or ^‡^ *P* < 0.01 versus Ad-Nox4 with VEGF. (c) Tube formation capacity of HRECs was evaluated by Matrigel assay. (c-A) Ad-LacZ, (c-B) Ad-Nox4, (c-C) Ad-Nox4 with PEG-Cat, and (c-D) quantification of tube numbers from 3 visual fields. Results represent 3 independent experiments. ^**^ *P* < 0.01 versus Ad-LacZ; ^†^ *P* < 0.05 versus Ad-Nox4.

To determine if Nox4's effect on angiogenic response is mediated by ROS, we treated Ad-Nox4-transduced HRECs with PEG-Catalase, a H_2_O_2_ scavenger, and then exposed the cells to VEGF to induce VEGFR2 activation. Consistent with previous observation, VEGF-induced VEGFR2 phosphorylation was augmented in HRECs overexpressing Nox4 (Figure 5(a)). This effect was completely abolished by PEG-Catalase. Interestingly, PEG-Catalase did not alter VEGF-induced VEGFR2 phosphorylation in cells transduced with Ad-LacZ. Similarly, PEG-Catalase did not show any effect on VEGF-induced ERK phosphorylation in control HRECs; however, it significantly attenuated Nox4's effect on ERK phosphorylation (Figure 5(b)). Next, we examined the role of H_2_O_2_ in Nox4-induced retinal endothelial tube formation. As shown in Figure 5(c), pretreatment with PEG-Catalase significantly suppressed endothelial tube formation induced by Nox4. These data indicate that Nox4's effect on endothelial angiogenic response is ROS/H_2_O_2_-dependent.

### 3.5. Knockdown of Nox4* In Vivo* Attenuates Retinal Neovascularization in Retinas of OIR Mice

**Figure 6:**
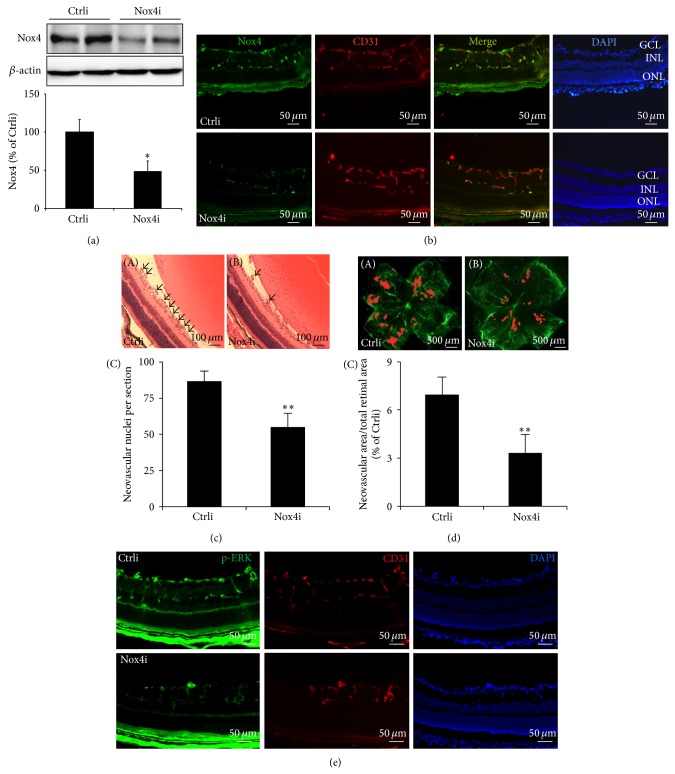
Knockdown of Nox4 ameliorates retinal NV in OIR mice. Ad-Ctrli and Ad-Nox4i were periocularly delivered to OIR mice at P12. (a-b) Retinal expression of Nox4 was determined by Western blot analysis at P15 (a) and immunostaining (b) at P17. *n* = 3 for each group; ^*^ *P* < 0.05 versus Ctrli. (b) Upper panel: Ad-Ctrli injected group and lower panel: Ad-Nox4i injected group. (c-d) Retinal NV formation was evaluated at P17 by counting nuclei of pre-ILM vasculature after HE staining (c) and by retinal angiography (d). HE staining: (c-A) retina from Ad-Ctrli injected mouse, (c-B) retina from Ad-Nox4i injected mouse, and (c-C) quantification result from 6 different mice in each group. Retinal angiography (d-A) retina from Ad-Ctrli injected mouse, (d-B) retina from Ad-Nox4i injected mouse, and (d-C) quantification result from 5 different mice in each group. ^*^ *P* < 0.05 or ^**^ *P* < 0.01 versus Ctrli. (e) Phosphorylation of ERK (green) and its colocalization with endothelial marker CD31 (red) were evaluated by immunostaining in P17 retinas. Images were representative of 4 different mice.

To determine if the effect of Nox4 on retinal NV also operates* in vivo*, we knocked down Nox4 gene in mouse retinas using Ad-Nox4i. Specifically, Ad-Nox4i was administrated into periocular compartment of OIR mice at P12, and retinal NV formation was evaluated by counting preretinal endothelial nuclei or retinal angiography at P17. Our results show that Ad-Nox4i reduced retinal Nox4 expression in P15 and P17 mice, determined by Western blot analysis (Figure 6(a)) and immunostaining (Figure 6(b)), respectively. Moreover, knockdown of Nox4 expression significantly suppressed retinal NV analyzed by counting pre-ILM endothelial nuclei in retinal sections (Figure 6(c)) and by quantification of NV in retinal angiography (Figure 6(d)). Furthermore, knockdown of Nox4 markedly reduced ERK phosphorylation in retinas of OIR mice at P17 (Figure 6(e)). These results support a role of Nox4 in regulation of ERK activation and retinal NV formation in OIR.

## 4. Discussion

NADPH oxidase is a major source of ROS in the retina [22, 23]. Suppressing NADPH activity by apocynin reduces retinal NV in OIR, suggesting a potential role of NADPH oxidase in retinal angiogenesis [24]. However, how NADPH oxidase regulates the angiogenic response of retinal endothelial cells remains poorly understood. Previously we identified that Nox4 is the major Nox isoform in retinal endothelial cells and regulates VEGF expression [16]. In the present study, we attempted to elucidate the role of Nox4 in retinal angiogenesis and pathological NV formation. Consistent with our previous finding, we observed that in mouse retina Nox4 was predominantly expressed in retinal vasculature and colocalized with CD31, an endothelial cell-specific marker. Interestingly, in retinas from P12 mice, Nox4 expression was mainly localized in the deep layers of retinal capillaries but not to the superficial blood vessels. Moreover, very weak signals of Nox4 were detected in retinal blood vessels from P15 mice. The dynamic changes in Nox4 expression and localization with blood vessels led us to suspect a potential role of Nox4 in retinal vascular development. In mouse retinas (e.g., retinas from C57BL/6J mice which were used in the present study), the superficial layer of retinal blood vessels forms during the first week after birth while the deep and intermediate vascular networks are formed during the second and third postnatal weeks [25]. These deep and intermediate vascular plexus, also referred to as secondary vascular networks [26], are formed by angiogenesis with new blood vessels sprouting from the superficial capillaries [25]. Intriguingly, recent work by the Lutty group showed that lack of Nrf2, a bZIP transcription factor that binds to antioxidant response elements and regulates antioxidant enzymes, dramatically affects the deep vascular network formation [26]. Their study also suggests that the angiogenic process occurring in the secondary network formation may generate increased levels of oxidative stress and those endothelial cells in the deep network which have high metabolic demands during differentiation and migration may be more susceptible to oxidative insult. Increased Nox4 expression in retinal endothelial cells in the deep vascular networks as observed in our study may be a potential source of ROS generation during postnatal angiogenesis.

In active neovascularization phase of OIR, high levels of Nox4 were detected in retinal blood vessels as well as preretinal NV. This finding, in line with a recent report that Nox4 expression was higher in the P18 OIR retina [27], again suggests a role of Nox4 in retinal angiogenesis. In addition, Nox4 signals colocalized with increased immunostaining of phosphorylated ERK. Furthermore, overexpressing Nox4 enhances ERK phosphorylation and exacerbates VEGF-induced ERK activation. In contrast, reducing Nox4 expression by siRNA alleviates VEGF-elicited ERK activation in retinal endothelial cells and attenuates ERK phosphorylation in retinal blood vessels in OIR. Some previous studies have shown that inhibition of Nox4 expression suppresses endothelial cell proliferation in human microvascular endothelial cells likely through the ERK pathway [28, 29]. These results together suggest that Nox4 regulates ERK activation and the angiogenic activities of retinal endothelial cells.

In addition to Nox4, other Nox isoforms, that is, Nox2, Duox1, and Duox2, were found in mouse retinas [30] and in human retinal endothelial cells [16]. In OIR, Nox2 expression is upregulated and localized to retinal vasculature [24] or leukocytes [31] and is potentially involved in retinal NV formation. Recently, using a genetic approach, Wilkinson-Berka et al. reported that only mice with NOX1 deletion, but not NOX2 or NOX4, protected retinas from ischemia-induced retinal vasculopathy [32]. However, Zhang et al. found a significant Nox4-dependent preservation of myocardial capillaries after pressure load [33]. Together with our findings, Nox4 may be essential for normal retinal angiogenesis and could have some protection from hyperoxia-induced retinal vessel dropout. Thus, the possible cause of the discrepancy with previous finding may be that genetic deletion of Nox4 during vasoobliteration phase may exacerbate retinal avascularity in OIR mice at P12, which, in turn, could compromise its beneficial effect on retinal NV at P17. In our previous study, we have also shown that depletion of Nox4 upregulates Nox2 but not Nox1 expression in retinal endothelial cells, suggesting a reciprocal regulation among Nox isoforms. Whether these isoforms, for example, Nox2 and Nox4, function synergistically to regulate endothelial angiogenic signaling remains to be investigated.

VEGF is a central regulator of retinal angiogenesis. We previously reported that hypoxia increases VEGF expression through upregulation of Nox4 in retinal endothelial cells and that inhibition of Nox4 attenuated retinal vascular leakage in db/db mice [16]. In the present study, we showed that knockdown of Nox4 also decreases retinal VEGF level in OIR (data not shown). In addition, overexpressing Nox4 potentiates VEGF-stimulated VEGFR2 activation in human retinal endothelial cells and enhances the cells' angiogenic responses (migration and tube formation) to VEGF. These effects appear to be mediated by ROS, as overexpressing Nox4 increased both intracellular [16] and extracellular ROS generation, and incubation with the H_2_O_2_ decomposer catalase almost completely abolished Nox4-induced enhancement in endothelial cell angiogenic activities. In contrast, scavenging H_2_O_2_ did not show any effect on VEGF-induced VEGFR2 phosphorylation, which is in agreement with a previous study [34]. The mechanisms by which Nox4-derived H_2_O_2_ enhances VEGFR2 activation are not fully understood. Previous study suggests that extracellular H_2_O_2_ generated by extracellular SOD (ecSOD) induces oxidation and inactivation of protein tyrosine phosphatase PTP1B, which negatively regulates VEGFR2 tyrosine phosphorylation in caveolae/lipid rafts [35]. Notably, we found that overexpression of Nox4 induced an over sixfold increase in extracellular ROS generation in retinal endothelial cells. Thus Nox4-generated H_2_O_2_ may also result in inactivation of PTP1B, as reported by Chen and associates [19], and promotes VEGFR2 activation. Future studies are needed to elucidate the role of PTP1B in retinal angiogenesis and NV formation in OIR.

Previous studies revealed that Nox4 expression is upregulated by multiple stressors such as hypoxia [36], hyperoxia [37], ischemia [13, 14, 38], and growth factors such as TGF-*β* and angiotensin II [39]. We observed increased Nox4 expression in OIR at both the vasoobliteration phase (i.e., P12) and the neovascularization phase (i.e., P15 and P17). This suggests that upregulation of Nox4 could be caused by hyperoxia as well as hypoxia. As hyperoxia exposure overlaps with the phase of physiologic vertical sprouting of vessels from the superficial layer into the deep and intermediate plexus, a marked delay in the formation of deeper retinal vasculature is expected in P12 OIR retina [25]. Indeed, in our study we observed significantly reduced amount of deep vascular plexus in P12 OIR retinas. This reduction in deep layers of blood vessels leads to decreased overall Nox4 level in the retina when quantified by Western blotting and real-time RT-PCR, which can be normalized by CD31 content. Thus, we speculate that Nox4 is not only involved in hypoxia-induced retinal angiogenesis, but also responsible for hyperoxia-induced retinal vessel dropout (vasoobliteration) in OIR. This hypothesis will be tested in our future studies.

In summary, our data indicates that upregulation of Nox4 promotes retinal neovascularization through ROS-dependent regulation of VEGF/VEGFR2 signaling pathway in OIR. Scavenging ROS by catalase or genetic inhibition of retinal Nox4 expression significantly blocked retinal endothelial angiogenic-related response* in vitro* and attenuated retinal neovascularization* in vivo*. Taken together, these results suggest that Nox4 plays a causal role in retinal angiogenesis and inhibition of Nox4 may provide a novel therapeutic strategy for neovascular retinal diseases.
